# Supplementary material for: Effects of Protein Supplementation Combined with Resistance Exercise Training on Walking Speed Recovery in Older Adults with Knee Osteoarthritis and Sarcopenia
Source: Nutrients. 2023 Mar 23;15(7):1552. doi: 10.3390/nu15071552 (PMC10096553; doi:10.3390/nu15071552)
Supplement: Supplementary file 1 [file nutrients-15-01552-s001.zip › nutrients-2290020-supplementary.pdf]

Table S1. Exercise progression protocol

Intensity: level of resistance (Theraband color)	Time progression (week)											
	1st	2nd	3rd	4th	5th	6th	7th	8th	9th	10th	11th	12th
	Supervised exercise training		Home-based exercise training									
Yellow	X	X										
Red			X	X								
Green					X	X						
Blue							X	X				
Black									X	X		
Silver											X	X
Exercise Loading												
Repetition	10–20	10–20	10–20	10–20	10–20	10–20	10–20	10–20	10–20	10–20	10–20	10–20
Set	3–5	6-10	3–5	6-10	3–5	6-10	3–5	6-10	3–5	6-10	3–5	6-10
RPE[a]	10–13	10–13	10–13	10–13	10–13	10–13	10–13	10–13	10–13	10–13	10–13	10–13

"X" denoted the intensity of resistance which is determined by Theraband color.
[a]Ratings of perceived exertion (RPE) using the Borg scale

Table S2. Elastic resistance exercise regime

Movement	Intensity (Repetition/Set)	Targeted muscle group	Duration (Min)
**A. Warm-up**			
1. Mobility exercise of the neck, upper limbs, and back		Upper quarter flexors and extensors	5
2. Global flexion-extension of the lower limb		Lower quarter flexors and extensors	5
**B. Upper quarter**			
1. Seated chest press	10–20/3	Upper quarter extensors	5–10
2. Seated row	10–20/3	Upper quarter flexors	5–10
3. Seated shoulder press	10–20/3	Shoulder girdle muscle groups	5–10
**C. Lower quarter**			
1. Concentric–eccentric hip circumduction	10–20/3	Hip girdle muscle groups	5–10
2. Leg press	10–20/3	Lower quarter extensors	5–10
3. Leg curl	10–20/3	Lower quarter flexors	5–10
**D. Cool down**			
1. Gentle stretching exercise<br>- Arm stretch<br>- Chest stretch<br>- Core stretch		Upper quarter flexors and extensors	5
2 Gentle stretching exercise<br>- Standing quad stretch<br>- Seated single-leg hamstring stretch<br>- Unilateral knee-to-chest		Lower quarter flexors and extensors	5
